# Annotations

**Published:** 1906-11-24

**Authors:** 


### ANNOTATIONS.

### Out-Patients and the Visiting Staff.

The problem presented by the ever-growing stream of out-patients to hospitals has received most attention from the point of view of hospital efficiency and the prevention of abuse. This, of course, is perfectly proper, since these considerations are paramount. But it will be readily understood that the conditions under which the out-patient staffs of hospitals conduct their work are seriously modified by the unwieldy proportions which their *clientèle* is assuming. Out-patient staffs are drawn from the ranks of junior consultants, except in the case of the large teaching hospitals, where the slowness of promotion conspicuously operates. These gentlemen accept the onerous duties of out-patient service, which implies in most cases an expenditure of two half-days a week, in order to gain knowledge, for the remuneration is either nothing or nominal. But their opportunites of acquiring knowledge are gravely compromised when the number of new patients to be dealt with exceeds, let us say, fifteen at one sitting. Where larger numbers present themselves the task of the doctor becomes little more than hack-work, shorn both of interest and efficiency. The last few years have witnessed a sensible diminution in the competition for such out-patient appointments, and should this tendency prove to be progressive, a further difficulty of no mean proportions will confront the managers of the smaller hospitals. We are writing from the standpoint of an experienced member of the visiting staff who realises, that, in practice, the ever-increasing number of new cases to be dealt with makes it impossible, at times, that a high standard of conscientious medical work can be maintained. This aspect has been largely lost sight of in considering the problem of hospital abuse. It is of the first importance however, and goes far to prove, that, the present system of excessive free medical relief must be drastically dealt with without delay.

### Clinical Medicine in the Seventeenth Century.

In his first FitzPatrick lecture, Dr. Norman Moore makes some interesting comments on the establishment of medical science in this country, and concludes that to the work of Mayerne, Glisson, and Sydenham the origin of the study of clinical medicine in England is mainly due. They are the three clinical observers of the seventeenth century whose work deserves the first place. While all three devoted themselves to bedside observation, Glisson was, in addition, a morbid anatomist. His mind seemed to turn most naturally to the discovery of pathological laws and to questions of etiology. Mayerne and Sydenham, on the other hand, were chiefly occupied with the solution of problems of treatment and of prognosis. All three observed carefully the general aspect of the patient and all the external features of the body; the breathing, the character of the pulse, the state of the tongue, the locality of pain, the indications of fever, the excreta and the appearances of extracted blood were considered. Tumours were felt, and the degree of dropsy estimated. Any impairment of the senses or of muscular power was noted. The liver and the spleen were examined by palpation. The history was carefully considered, and facts bearing on heredity were recorded. This statement summarises the extent to which observation at the bedside was practised by these physicians. Of the three, Mayerne seems most in personal relation to the patient, while Glisson is most considerate of the interpretation of symptoms given by the morbid anatomy. Sydenham had always before him the endeavour to establish general laws in relation to disease, and hoped to do so by a precision of description such as that practised by the botanists in the description of plants. The whole of Dr. Moore's lecture may be read with advantage and interest, and the busy practitioner who has little time for historical and literary research may consult it with pleasure, and with a feeling of gratitude both to the lecturer and to the generous donor whose enlightened liberality made the FitzPatrick foundation possible.

### The Right Hon. Robert Farquharson, M.D.

The inhabitants of Aberdeenshire have with cordial unanimity testified their warm affection and esteem for the Right Hon. Robt. Farquharson, M.D., on the occasion of his retirement, after twenty-five years' service as M.P. for West Aberdeenshire. Dr. Farquharson has been a popular figure in Parliament and in London professional and society life for so many years, that his retirement from Parliament has been regretted by members of all parties, and indeed by all who take the most active interest in the advancement of the social life and well-being of the people. Dr. Farquharson has had a long and varied professional experience. He has been a surgeon in the Guards, a member of the visiting staff of St. Mary's Hospital and a lecturer in its medical school, and physician to the Western General and Chelsea Dispensaries. He was formerly medical officer to Rugby School. In all these varied capacities Dr. Farquharson rendered good service, and always proved a popular force and a pleasant colleague. He was actively associated with the institution of the Sanitary Institute of Great Britain, in the development of which he took a great interest, and his writings and speeches show that public hygiene and health are subjects the importance of which he was the first to recognise. He has written upon school hygiene and the diseases incidental to school life on which he was an authority. Born in 1836, and graduating at Edinburgh University in 1858, Dr. Farquharson has rendered much useful public service. Sir John Clarke, who made the presentation on behalf of all shades of political opinion in Aberdeenshire, declared that Dr. Farquharson was one of the best-beloved men in the whole country. Few men spend as many years as Dr. Farquharson has spent in public life and share his good fortune by securing at the end of such service the unanimous and grateful recognition of those who have been opposed to him in politics. That Dr. Farquharson should occupy this proud position is the best evidence of his popularity and of the excellent spirit which has characterised all his actions throughout a long and active career of public and professional usefulness.

